# Catheter ablation for patients with anomalous pulmonary venous return and atrial fibrillation: a case report and literature review

**DOI:** 10.1093/ehjcr/ytae292

**Published:** 2024-06-10

**Authors:** Zhaoyang Wei, Minghua Li, Zhenggui Wang, Zhiguo Zhang

**Affiliations:** Department of Cardiology, The First Hospital of Jilin University, Changchun, Jilin Province 130021, China; Department of Cardiology, The First Hospital of Jilin University, Changchun, Jilin Province 130021, China; Department of Cardiology, The First Hospital of Jilin University, Changchun, Jilin Province 130021, China; Department of Cardiology, The First Hospital of Jilin University, Changchun, Jilin Province 130021, China

**Keywords:** Anomalous pulmonary venous return, Atrial fibrillation, Catheter ablation, Case report

## Abstract

**Background:**

Anomalous pulmonary venous return involves the partial or complete absence of a connection between the pulmonary veins and the left atrium. The pulmonary vein potential plays a vital role in atrial fibrillation, and catheter ablation to isolate the pulmonary vein is crucial for treating this condition. However, when anomalous pulmonary venous return is present, it makes ablation more challenging and increases the risk of atrial fibrillation recurrence after the procedure.

**Case summary:**

A 49-year-old man was hospitalized because he had been experiencing occasional palpitations for 2 months. He had previously undergone surgery to repair an atrial septal defect when he was 11 years old, during which an issue with the right inferior pulmonary vein was identified but left unaddressed. Electrocardiography upon admission showed atrial fibrillation. Left atrial computed tomography angiography revealed that following atrial septal repair surgery, the right inferior pulmonary vein drained into the right atrium. The patient underwent transcatheter radiofrequency ablation to electrically isolate the pulmonary vein with anomalous return. After 12 months of follow-up, there was no atrial fibrillation recurrence.

**Discussion:**

When performing catheter ablation for anomalous pulmonary venous return and atrial fibrillation, it is essential to consider ablating the irregular pulmonary vein before surgery. This helps to reduce surgical complications and the likelihood of atrial fibrillation recurrence. This case report highlights the challenges encountered during ablation in patients with atrial fibrillation and anomalous pulmonary venous return. In addition, we have reviewed the literature to offer insights into the development of ablation strategies for similar patients.

**Learning points**

- Abnormal pulmonary vein connections near critical structures can cause atrial fibrillation, and while ablation carry risks, not performing ablation may lead to atrial fibrillation recurrence.
- When considering ablation of anomalous pulmonary veins, the possibility of damaging surrounding tissues should be taken into account.

## Introduction

Anomalous pulmonary venous return (APVR) is a rare congenital heart disease comprising total anomalous pulmonary venous return (TAPVR) and partial anomalous pulmonary venous return (PAPVR).^1, 2^ Unfortunately, most individuals with TAPVR do not survive into adulthood.^3^ Patients diagnosed with PAPVR typically exhibit no symptoms and have at least one pulmonary vein that does not connect to the left atrium.^4^ Notably, the pulmonary veins play a critical role in the onset and maintenance of atrial fibrillation.^5^ Consequently, pulmonary vein isolation is crucial for catheter ablation when treating atrial fibrillation.^6^ In atrial fibrillation cases accompanied by PAPVR, where the pulmonary veins do not connect to the left atrium, there often exist challenging anatomical relationships with structures like the phrenic nerve and oesophagus. These complexities significantly complicate catheter ablation procedures. Therefore, it is imperative to explore catheter ablation treatment strategies that are specifically tailored to patients with APVR and atrial fibrillation.

## Summary figure

**Figure:**
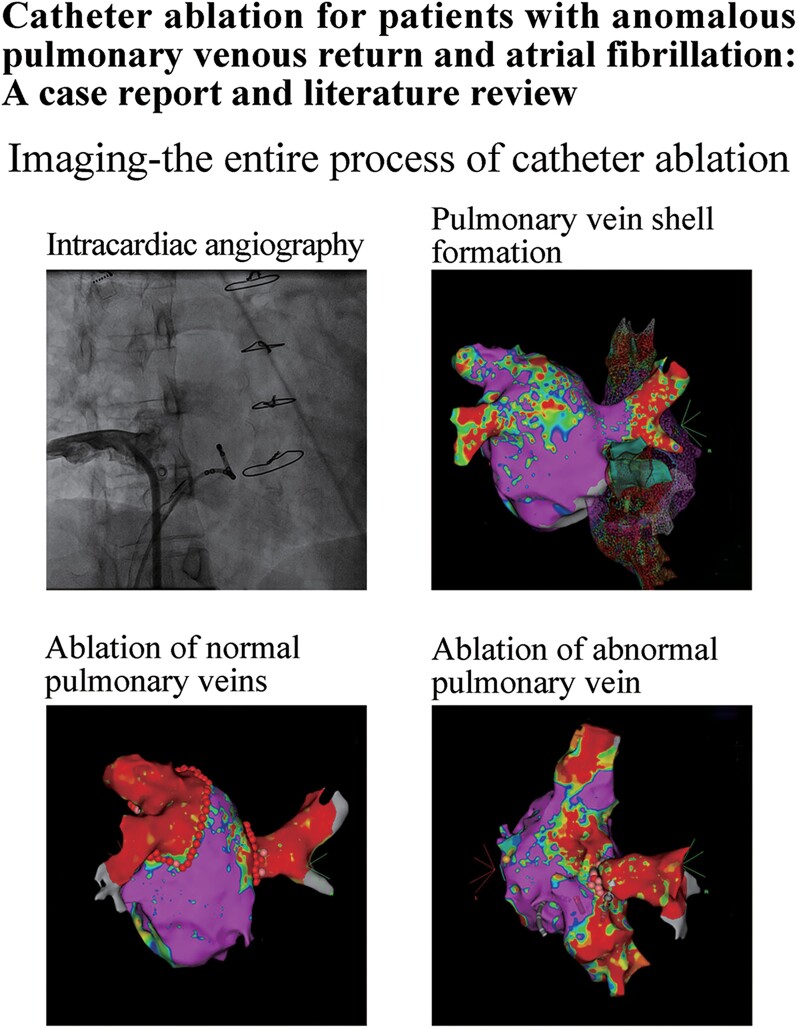


## Case presentation

**Figure 1:**
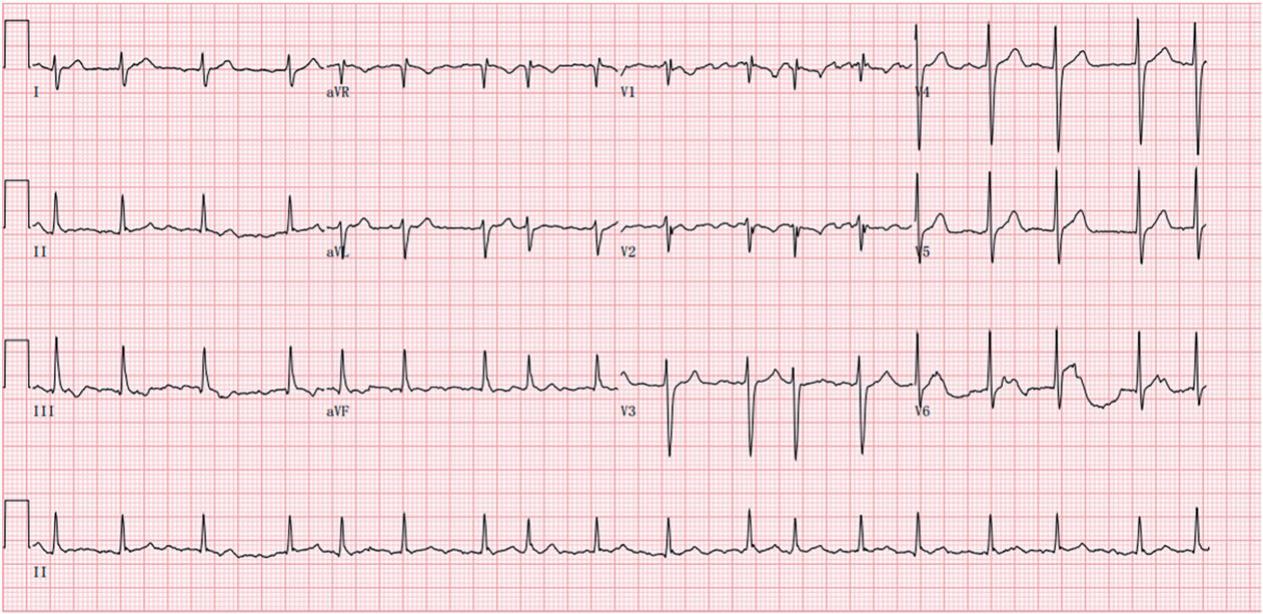
The pre-ablation electrocardiogram indicated atrial fibrillation.

A 49-year-old male was admitted to our hospital with a history of intermittent palpitations over the previous two months and was found to be in atrial fibrillation (*Figure 1*). Notably, the patient had previously undergone atrial septal defect repair surgery at the age of 11 years. During the surgery, it was noted that the right inferior pulmonary vein was abnormally connected to the right atrium, but this abnormality was not surgically addressed. Unfortunately, the patient was unable to provide any details on the type of atrial septal defect, and the surgical details. Upon admission, physical examination revealed irregular intensity of heart sounds and pulse irregularities, indicating the presence of atrial fibrillation in the patient. The patient’s blood test results did not show any abnormalities. The echocardiogram revealed a left atrium size of 42 × 50 × 54 mm, right atrium size of 48 × 48 mm, ejection fraction of 63%, and a patch was observed in the atrial septum. Computed tomography angiography of the left atrium revealed a history of atrial septal defect repair surgery in the patient, along with the identification of right inferior pulmonary vein draining into the right atrium. Prior to admission, the patient had been taking metoprolol orally, which showed poor efficacy in alleviating palpitation symptoms. After discussing treatment options with the patient, he declined trying other antiarrhythmic drugs and opted for catheter ablation therapy.

**Figure 2:**
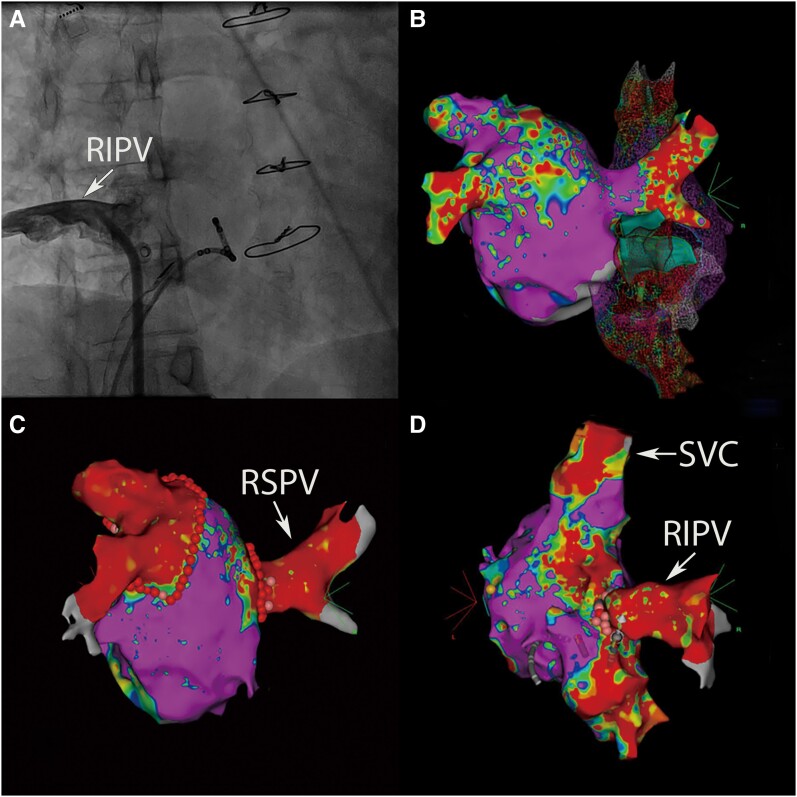
(*A*) Intracardiac angiography displays the connection of the right inferior pulmonary vein to the right atrium. (*B*) Anatomical illustrations of both the left and right atria generated using the CARTO system. (*C*) Isolation of the normally connected pulmonary veins. (*D*) Isolation of the abnormally connected right inferior pulmonary vein. RIPV, right inferior pulmonary vein; RSPV, right superior pulmonary vein; SVC, superior vena cava.

Upon arrival in the catheterization room, the patient underwent standard pre-operative preparation. After completing the pre-operative measures, local anaesthesia with 1% lidocaine was administered. Subsequently, catheter placement ensued, involving the insertion of an 11 F sheath (APT Medical, China, 1 F ≈ 0.33 mm) into the left femoral vein, a 7 F sheath (APT Medical) and an SL1 long sheath (Synaptic Medical, China) into the right femoral vein. The coronary sinus electrode (APT Medical) was inserted into the 7 F sheath, and the intracardiac ultrasound catheter (Biosense Webster, USA) was placed into the 11 F sheath. The intracardiac ultrasound catheter was applied for the preliminary mapping of the left atrium and pulmonary veins. After that, contrast agent was injected into the right atrium via the SL1 long sheath. This procedure confirmed that the right inferior pulmonary vein drained into the right atrium, consistent with atrial computed tomography angiography findings (*Figure 2A*). Subsequently, under intracardiac ultrasound guidance, the septal puncture needle (Synaptic Medical) traversed the SL1 long sheath, successfully puncturing the interatrial septum. The PENTARAY catheter (Biosense Webster) was also introduced through the SL1 long sheath in the right femoral vein to map the left atrium and pulmonary veins (*Figure 2B*). During mapping, electrical activity was identified within the pulmonary vein with anomalous return. Following completion of mapping, the CARTO Smart Touch ablation catheter (Biosense Webster) was introduced to perform radiofrequency ablation (45 W power, ablation index 450) on the normally connected pulmonary veins (*Figure 2C*). Upon pacing the right inferior pulmonary vein with the ablation catheter, no diaphragmatic twitching was observed, confirming that the catheter was not adjacent to the phrenic nerve. Subsequently, while pacing the right phrenic nerve, ablation (35 W power, ablation index 400) was delivered to the ostium of the anomalous right inferior pulmonary vein to isolate the vein (*Figure 2D*). The ablation points effectively encircled each pulmonary vein. Post-ablation, the PENTARAY catheter was employed to assess each pulmonary vein, confirming the absence of pulmonary vein potentials. Following completion of ablation, standard electrophysiological testing was conducted, which did not induce any other arrhythmias. Whereafter, the patient safely returned to the ward. The patient was discharged 2 days after catheter therapy. In accordance with ESC guidelines,^7^ the patient receives rivaroxaban 20 mg orally once daily (for three months), amiodarone 200 mg orally (three times daily for one week, twice daily for one week, and once daily until three months post-operatively). During a 12-month follow-up, no atrial fibrillation recurrence was observed.

## Discussion

Anomalous pulmonary venous return is a rare structural defect that is frequently accompanied by other cardiac abnormalities.^8^ In patients with APVR, the pulmonary veins are often connected to the right atrium or superior vena cava. Recent research has indicated that the heart muscle extending from the pulmonary vein to the left atrium can increase the likelihood of atrial fibrillation.^9^ However, in patients with atrial fibrillation and APVR, irregular pulmonary veins may trigger atrial fibrillation through various mechanisms. Moreover, anomalous pulmonary vein connections may be in close proximity to important anatomical structures such as major arteries, the oesophagus, and the phrenic nerve, posing a risk of injuring surrounding tissues during pulmonary vein isolation. In this case, there was concern that the anomalous pulmonary vein may be adjacent to the phrenic nerve. We confirmed the safety to ablate without damaging the phrenic nerve by first pacing around the anomalous pulmonary vein to confirm no phrenic nerve capture, and then additionally by simultaneously pacing the phrenic nerve whilst ablating around the anomalous pulmonary with no loss of capture during ablation. Due to the intricate cardiac structure in patients with congenital heart disease, there is a relatively high rate of atrial fibrillation recurrence after ablation.^10^ However, there is no agreement on whether or how to ablate irregular pulmonary veins. Some studies have reviewed the medical records of patients similar to ours before 2022.^4^ We have supplemented this information (*Table 1*). To date, adding to the number of cases found in previous research,^4^ we have identified 24 male-predominant cases of atrial fibrillation catheter ablation in patients with APVR. Among these cases, five experienced atrial fibrillation recurrence (three with pre-existing APVR electrical activity and two without). None of the cases with atrial fibrillation recurrence underwent electrical isolation of APVR.^4^ On the contrary, in cases where electrical potentials were present in APVR and isolation was performed, no instances of atrial fibrillation recurrence were reported.^4^ Based on these findings, we posit that the benefits of routinely performing electrical isolation on APVR outweigh the risks. This case report provides the following prompts. Firstly, if considering ablation therapy for atrial fibrillation in APVR patients, computed tomography, magnetic resonance imaging, and similar methods should be used to assess structures near to the irregular pulmonary veins (e.g. the phrenic nerve, oesophagus, and major arteries) to gauge the ablation complexity. Secondly, when electrical activity is identified in anomalous connected pulmonary vein, it is advisable to isolate that pulmonary vein. Finally, if the irregular pulmonary vein lacks a clear activation signal and is closely connected to other structures, the ablation risk is high, and it may not be suitable to isolate the vein.

**Table 1:** Demographic, anatomical, and procedural data of published cases (supplement to prior research)

First author	Journal, year of publication	Sex	Age (y)	APVR	APVR connection	Imaging modality	APVR electrical activity	EAM	Energy	APVR ablation	Follow-up (mo)	Recurrence (mo)
Patel *et al*.^11^	Circ Arrhythmia Electrophysiol, 2008	M	41	RIPV, LIPV	Common ostium in left atrium	CT	Y	Carto	RF	N	3	N
Kirubakaran *et al*.^12^	Circ Arrhythm Electrophysiol, 2013	M	25	TAPVR	Unroofed coronary sinus	CT	Y	Carto	RF	Y (the entire venous confluence)	9	N
Bhalla *et al*.^13^	Europace, 2022	M	72	RSPV	SVC	CT	N	Carto	RF	N	6	N
Xiang and Wei^14^	Heart Surg Forum, 2022	M	45	TAPVR	Innominate vein	CT	NA	NA	RF	Y (right atrium to the inferior vena cava)	24	N
Milstein *et al*.^15^	J Am Coll Cardiol, 2023	F	56	RSPV	SVC	CT	NA	NA	CT + RF	Y (SVC isolation)	5	N

APVR, anomalous pulmonary venous return; CT, computed tomography; EAM, electro anatomical mapping; LIPV, left inferior pulmonary vein; NA, not available; RF, radiofrequency ablation; RIPV, right inferior pulmonary vein; RSPV, right superior pulmonary vein; SVC, superior vena cava; TAPVR, total anomalous pulmonary venous return.

## Lead author biography


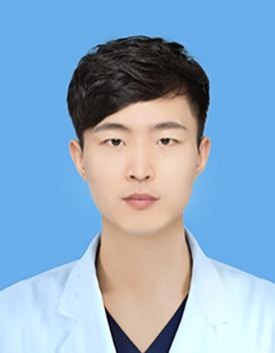


Zhaoyang Wei graduated from Jilin University and is a junior physician at the First Hospital of Jilin University. His current interests include interventional cardiology, the integration of artificial intelligence and medicine.

## Data Availability

All data are available from the corresponding author.
